# Interaction between Maternal Passive Smoking during Pregnancy and CYP1A1 and GSTs Polymorphisms on Spontaneous Preterm Delivery

**DOI:** 10.1371/journal.pone.0049155

**Published:** 2012-11-13

**Authors:** Yi-Juan Luo, Xiao-Zhong Wen, Peng Ding, Yan-Hui He, Chuan-Bo Xie, Tao Liu, Jian-miao Lin, Shi-Xin Yuan, Xiao-Ling Guo, De-Qin Jia, Li-Hua Chen, Bao-Zhen Huang, Wei-Qing Chen

**Affiliations:** 1 Department of Biostatistics and Epidemiology, School of Public Health, Sun Yat-sen University, Guangzhou, Guangdong, China; 2 Obesity Prevention Program, Department of Population Medicine, Harvard Medical School and Harvard Pilgrim Health Care Institute, Boston, Massachusetts, United States of America; 3 Shenzhen Women and Children’s Hospital, Shenzhen, Guangdong, China; 4 Foshan Women and Children’s Hospital, Foshan, Guangdong, China; Gentofte University Hospital, Denmark

## Abstract

**Objective:**

The present study aimed to examine the association between maternal passive smoking during pregnancy and the risk of spontaneous PTD and to explore the potential interaction of the single or joint gene polymorphism of CYP1A1 and GSTs with maternal passive smoking on the risk of spontaneous PTD.

**Method:**

We investigated whether the association between maternal passive smoking and PTD can be modified by 2 metabolic genes, i.e. cytochrome P4501A1 (CYP1A1) and glutathione S-transferases (GSTs), in a case-control study with 198 spontaneous preterm and 524 term deliveries in Shenzhen and Foshan, China. We used logistic regression to test gene-passive smoking interaction, adjusting for maternal socio-demographics and prepregnancy body mass index.

**Results:**

Overall, maternal passive smoking during pregnancy was associated with higher risk of PTD (adjusted odds ratio = 2.20 [95% confidence interval: 1.56–3.12]). This association was modified by CYP1A1 and GSTs together, but not by any single genotype. For cross-categories of CYP1A1 Msp I and GSTs, maternal passive smoking was associated with higher risk of PTD among those women with CYP1A1 “TC/CC”+ GSTs “null”, but not among women with other genotypes; and this interaction was significant (OR = 2.66 [95% CI: 1.19–5.97]; P-value: 0.017). For cross-categories of CYP1A1 BsrD I and GSTs, maternal passive smoking was associated with higher risk of PTD only among those women with CYP1A1“AG/GG”+ GSTs “null”, but not among women with other genotypes; and this interaction was significant (OR = 3.00 [95% CI: 1.17–7.74]; P-value: 0.023).

**Conclusions:**

Our findings suggest that the combined genotypes of CYP1A1 and GSTs can help to identify vulnerable pregnant women who are subject to high risk of spontaneous PTD due to passive smoking.

## Introduction

Preterm delivery (PTD, <37 completed weeks of gestation) is a big clinical and public health challenge globally. Despite increased awareness and improved prenatal care, the percentage of PTD remains unacceptably high in both developed (e.g. 7% in U.S. [1]) and developing societies (e.g. 5–15% in China [2]). About 15% preterm babies die within one month after birth [3]. PTD also leads to many other short- and long-term health problems and poses enormous burden to both health care system and the child’s family [4]. Preterm delivery is often classified into spontaneous and medically indicated subgroups, and the majorities (75–85%) of PTDs are spontaneous [5]. The causes and underlying biological mechanisms of spontaneous PTD are still unclear [6], although previous studies have identified a long list of risk factors, including low socio-economic status [7], parity, maternal age [8], drug abuse, life events [9], racial origin [10], maternal active or passive smoking [11], air pollution [12], [13] intrauterine infection [14], and genetic heterogeneity [9], [15].

Maternal active smoking is a well-established risk factor for PTD, and it accounts for about 14% of all PTDs [16]. But only a relatively small proportion of smoking pregnant women end with PTD, which may be explained by the substantial variability in genetic susceptibility across individuals. Some evidences show that gene polymorphism in cytochrome P4501A1 (CYP1A1) and glutathione S-transferases (GSTs) may modify the link between maternal active smoking and PTD [17]. CYP1A1 is one of cytochrome P450 (CYP450) family genes that are responsible for phase I detoxication by converting exogenous exposures, e.g. tobacco compounds, into intermediate metabolites. CYP1A1 mutation can lead to higher enzyme activity. GSTs are one of phase II detoxication enzymes that protect cells from toxicants by conjugation with glutathione. Inherited homozygous deletion of GSTs genes can result in lack of phase II detoxification activity [18], [19], [20], which thus increases the accumulation of intermediate metabolites of exogenous in human body. Limited researches focused on the gene (CYP1A1, GSTs)-maternal active smoking on the risk PTD yielded inconsistent findings [17], [21], [22].

**Figure 1:**
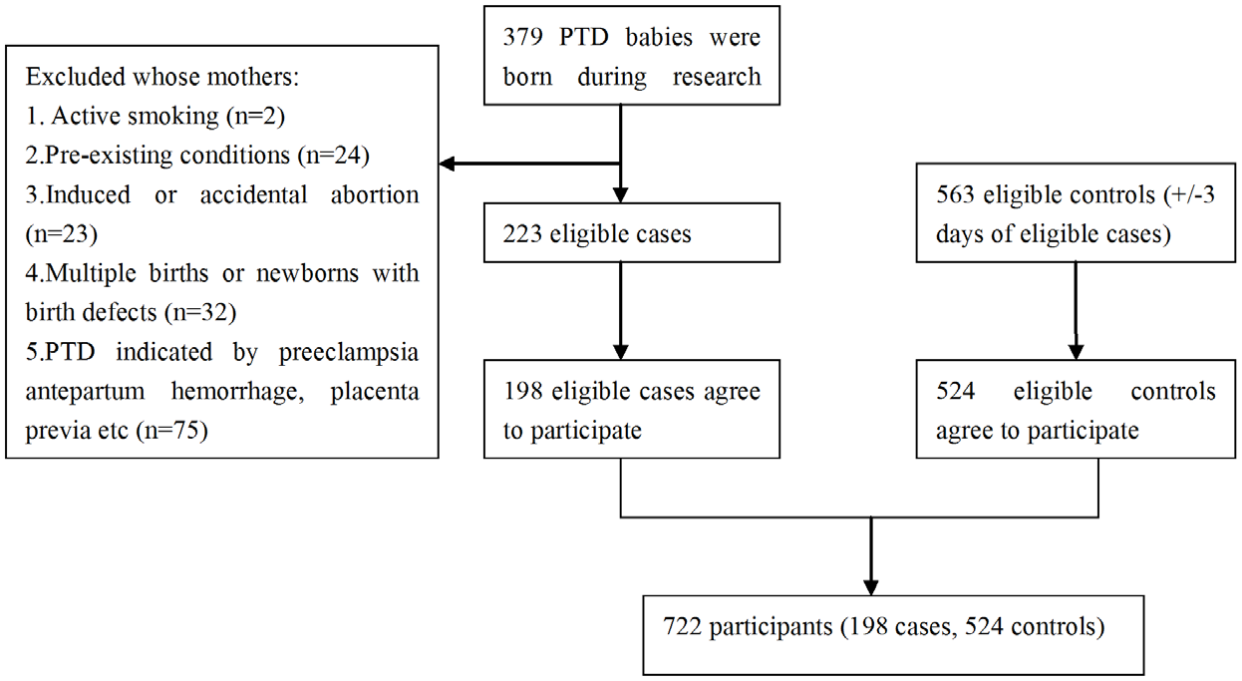
Flow chart of study participants.

Passive smoking is one of the most important public health problems in many developing countries, such as China, where many non-smoking pregnant women are exposed to environmental tobacco smoke at home, workplace, and public places [23]. Perera et al. found the levels of serum cotinine and DNA adducts in passive smoking mothers were significantly higher than those in non-passive smoking mothers [24]. Some previous studies, all done in developed countries, suggest that maternal passive smoking may shorten gestational age and thus increase the risk of PTD [23]. It is unclear whether this association holds in pregnant women in developing countries. Moreover, little is known about the potential interaction between maternal passive smoking and genes (CYP1A1, GSTs) polymorphisms in the risk of PTD.

**Table 1:** Comparison of characteristics between spontaneous PTD cases and controls.

	Cases (n = 198)	Controls(n = 524)	P-value
	Mean ± SD or N. (%)	Mean ± SD or N. (%)	
Maternal age (years, mean ± SD)	28.5±4.9	28.8±4.3	0.474
Marital status (%)			
Married	193(97.5)	502(95.8)	0.290
Unmarried	5(2.5)	22(4.2)	
Race/ethnicity (%)			
Han	191(97.0)	507(96.9)	0.992
Minority	6(3.0)	16(3.1)	
Education level (%)			
Junior high school or lower	70(35.4)	136(26.0)	0.020
High school	55(27.8)	142(27.1)	
College or higher	73(36.9)	246(46.9)	
Family income (%)			
Low	44(23.0)	141(27.2)	0.248
Middle	32(16.8)	102(19.7)	
High	115(60.2)	276(53.2)	
Parity (%)			
Nullparous	134(67.7)	398(76.0)	0.024
Parous	64(32.3)	126(24.0)	
Alcohol use during pregnancy, %	4(2.0)	17(3.2)	0.383
Prepregnancy BMI (kg/m^2^) (%)			
Underweight (<18.5)	45(22.7)	124(23.7)	0.606
Normal (18.5–23.9)	137(69.2)	346(66.0)	
Overweight or obesity(≥24.0)	16(8.1)	54(10.3)	
Child gender (%)			
Male	109(35.6)	257(51.3)	0.105
Female	89(64.4)	271(48.7)	

Therefore, the present study aimed to fill these important research gaps in this filed. Specially, we had 2 objectives in this study: 1) to examine the association between maternal passive smoking during pregnancy and the risk of spontaneous PTD among Chinese pregnant women; 2) to explore the potential interaction between the single or joint gene polymorphism of CYP1A1 and GSTs with maternal passive smoking on the risk of spontaneous PTD.

**Table 2:** Associations of maternal passive smoking during pregnancy, CYP1A1, and GSTs genotypes with risks of spontaneous PTD.

	Case (n = 198)	Control (n = 524)	Crude OR (95% CI)	OR^a^ (95%CI)	P-value
	N. (%)	N. (%)			
Passive smokingduring pregnancy					
No	101(51.0)	369(70.4)	1	1	
Yes	97(49.0)	155(29.6)	2.29 (1.63–3.20)^ *^	2.20 (1.56–3.12)	0.000
CYP1A1 Msp I					
TT	73(36.9)	212(40.5)	1	1	
TC/CC	125(63.1)	312(59.5)	1.16 (0.83–1.63)	1.15 (0.82–1.63)	0.418
CYP1A1 BsrD I					
AA	95(48.0)	290(55.3)	1	1	
AG/GG	103(52.0)	234(44.7)	1.34 (0.97–1.87)	1.30 (0.93–1.81)	0.133
GSTM1					
Present	97(49.0)	275(52.5)	1	1	
Null	101(51.0)	249(47.5)	1.15 (0.83–1.60)	1.12 (0.80–1.57)	0.515
GSTT1					
Present	95(48.0)	241(46.0)	1	1	
Null	103(52.0)	283(54.0)	0.92 (0.67–1.28)	0.97 (0.69–1.37)	0.877
GSTs					
Present	137(69.2)	386(73.7)	1	1	
Null	61(30.8)	138(26.3)	1.25 (0.87–1.78)	1.29 (0.89–1.87)	0.182

a. OR^a^ adjusted for family income, maternal age, education level and prepregnancy BMI.

b. GSTs “null” if both GSTM1 and GSTT1 “null”, GSTs “present” if either GSTM1 or GSTT1 “present”.

## Materials and Methods

### Study Population

We conducted a case-control study from September 2009 to March 2011 at two Women and Children’s Hospitals at Shenzhen and Foshan, Guangdong Province, China. Figure 1 shows the flow chart of our study participants flowchart was listed in figure 1. A total of 379 preterm (gestational age <37 full weeks) babies were born in these two hospitals during the study period. Among them, 223 spontaneous singleton babies were eligible for this study (see below). Finally, 198 (88.8%) mothers of 223 eligible babies agreed to participate in the study and were included as cases. We randomly selected controls from those mothers who delivered term singleton babies (gestational age 37–42 full weeks) with normal birth weight (2500–4000 g) in the same hospital. To increase the comparability, we matched 563 eligible controls with eligible cases by delivery date (+/−3 days). Among them, 524 (93.1%) mothers of the controls agreed to participate in this study. We approached participating mothers within 12–36 hours after delivery.

**Table 3:** Interaction between maternal passive smoking during pregnancy and single genotype of CYP1A1 and GSTs on risk of spontaneous PTD.

Passivesmoking	Genotype	Case(n = 198)	Control(n = 524)	OR^a^(95%CI)	P-value
		N.(%)	N.(%)		
	CYP1A1 Msp I				
No	TT	41 (20.7)	151 (28.8)	1	
No	TC/CC	60 (30.3)	218 (41.6)	0.96 (0.61–1.52)	0.876
Yes	TT	32 (16.2)	61 (11.6)	1.75 (1.00–3.07)*	0.052
Yes	TC/CC	65 (32.8)	94 (17.9)	2.42 (1.50–3.89)*	0.000
Interaction				1.44 (0.71–2.92)	0.319
	CYP1A1 BsrD I				
No	AA	50 (25.3)	203 (38.7)	1	
No	AG/GG	51 (23.5)	166 (31.7)	1.18 (0.75–1.85)	0.483
Yes	AA	45 (22.7)	87 (16.6)	1.98 (1.22–3.22)^*^	0.007
Yes	AG/GG	52 (26.3)	68 (13.0)	2.90 (1.78–4.72)^*^	0.000
Interaction				1.24 (0.62–2.47)	0.541
	GSTM1				
No	Present	49 (24.7)	195 (37.2)	1	
No	Null	52 (26.2)	174 (35.7)	1.15 (0.73–1.81)	0.519
Yes	Present	48 (24.2)	80 (15.3)	2.30 (1.41–3.74)	0.001
Yes	Null	49 (24.7)	75 (29.5)	2.43 (1.49–3.97)	0.000
Interaction				0.92 (0.46–1.83)	0.806
	GSTT1				
No	Present	49 (24.7)	163 (31.1)	1	
No	Null	52 (26.2)	206 (39.3)	0.84 (0.53–1.33)	0.454
Yes	Present	46 (23.2)	78 (14.9)	1.81 (1.10–2.98)	0.020
Yes	Null	51 (25.8)	77 (14.7)	2.22 (1.37–3.62)	0.002
Interaction				1.46 (0.73–2.91)	0.286
	GSTs				
No	Present	73 (36.9)	267 (51.0)	1	
No	Null	28 (14.1)	102 (19.5)	1.00 (0.60–1.66)	0.999
Yes	Present	64 (32.3)	119 (22.7)	1.86 (1.24–2.81)	0.003
Yes	Null	33 (16.7)	36 (6.9)	3.39 (1.95–5.91)	0.000
Interaction				1.83 (0.84–3.96)	0.126

a. OR^a^ adjusted for family income, maternal age, education level and prepregnancy BMI.

b. GSTs “null” if both GSTM1 and GSTT1 “null”, GSTs “present” if either GSTM1 or GSTT1 “present”.

c. % for distribution within the case and control groups respectively.

For the purpose of this study, we excluded the preterm babies whose mothers: 1) actively smoked cigarettes during pregnancy (n = 2); 2) had one or more pre-existing chronic conditions, including heart failure, chronic renal diseases, lung diseases, diabetes, hypertension, hyperthyroidism, Mediterranean anemia etc. (n = 24); 3) had induced or accidental abortion (n = 23); 4) had multiple births or newborns with birth defects (n = 32); or 5) had medically indicated PTD due to obstetric complications, such as severe preeclampsia, antepartum hemorrhage, placenta previa etc (n = 75).

**Table 4:** Interaction between maternal passive smoking during pregnancy and joint genotype of CYP1A1 Msp I and GSTs on risk of spontaneous PTD.

Passive smoking	Genotype		Case (n = 198)	Control (n = 524)	OR^a^(95%CI)	P-value
	CYP1A1 Msp I	GSTs	N. (%)	N. (%)		
No	TT	Present	30 (15.2%)	110 (21.0%)	1	
No	TT	Null	11 (5.6%)	41 (7.8%)	1.04 (0.47–2.29)	0.918
No	TC/CC	Present	43 (21.7%)	157 (30.0%)	0.98 (0.57–1.67)	0.947
No	TC/CC	Null	17 (8.6%)	61 (11.6%)	0.95 (0.47–1.93)	0.902
						
Yes	TT	Present	25 (12.6%)	45 (8.6%)	1.82 (0.95–3.48)	0.072
Yes	TT	Null	7 (3.5%)	16 (3.1%)	1.64 (0.61–4.41)	0.326
Yes	TC/CC	Present	39 (19.7%)	74 (14.1%)	1.85 (1.04–3.26)	0.035
Yes	TC/CC	Null	26 (13.1%)	20 (3.8%)	4.72 (2.28–9.77)	0.000
Interaction					2.66 (1.19–5.97)	0.017

a. OR^a^ adjusted for family income, maternal age, education level, prepregnancy BMI and CYP1A1 BsrD I genotype.

b. GSTs “null” if both GSTM1 and GSTT1 “null”, GSTs “present” if either GSTM1 or GSTT1 “present”.

c. % for distribution within the case and control groups respectively.

d .The variable of passive smoking+CYP1A1 Msp I+ GSTs with eight levels was set as dummy independent variable, and the first level as reference category.

**Table 5:** Interaction between maternal passive smoking during pregnancy and joint genotype of CYP1A1 BsrD I and GSTs on risk of spontaneous PTD.

Passive smoking	Genotype		Case (n = 198)	Control (n = 524)	OR^a^(95%CI)	P-value
	CYP1A1 BsrD I	GSTs	N. (%)	N. (%)		
No	AA	Present	38 (19.2%)	149 (28.4%)	1	
No	AA	Null	12 (6.1%)	54 (10.3%)	0.94 (0.45–1.95)	0.863
No	AG/GG	Present	35 (17.7%)	118 (22.5%)	1.14 (0.67–1.95)	0.636
No	AG/GG	Null	16 (8.1%)	48 (9.2%)	1.19 (0.59–2.42)	0.613
Yes	AA	Present	30 (15.2%)	61 (11.6%)	1.80 (1.01–3.22)	0.050
Yes	AA	Null	15 (7.6%)	26 (5.0%)	2.33 (1.11–4.89)	0.030
Yes	AG/GG	Present	34 (17.2%)	58 (11.1%)	2.17 (1.23–3.83)	0.008
Yes	AG/GG	Null	18 (9.1%)	10 (1.9%)	7.01 (2.91–16.86)	0.000
	Interaction				3.00 (1.17–7.74)	0.023

a. OR^a^ adjusted for family income, maternal age, education level, prepregnancy BMI and CYP1A1 Msp I genotype.

b. GSTs “null” if both GSTM1 and GSTT1 “null”, GSTs “present” if either GSTM1 or GSTT1 “present”.

c. % for distribution within the case and control groups respectively.

d. The variable of passive smoking+CYP1A1 BsrD I + GSTs with eight levels was set as dummy independent variable, and the first level as reference category.

All the participants understood and signed a written consent form. This study was approved by the Ethics Committees of Sun Yat-sen University in Guangzhou, China.

### Data Collection

We collected data through interview, medical records review, and blood lab tests. At the postnatal face-to-face interview, each mother completed a structural questionnaire and reported her passive smoking status during pregnancy, socio-demographics, reproductive history, medical history, psychosocial stress, health behavior and lifestyles. Pregnant women were expected to complete up to 12 routine prenatal care visits for routine obstetric examinations starting from the 20^th^ week of gestation: 2 visits during 20–27 weeks of gestation, bi-weekly during 28–35 weeks of gestation, and then weekly after 36 weeks of gestation. We obtained maternal and fetal health information from obstetrical medical records, including last menstrual period, ultrasound assessment, maternal chronic diseases, and obstetric complications. We collected maternal blood samples within 12 hours after admission to the hospital in tubes with anticoagulants (EDTA K2), and then stored blood samples in a refrigerator at temperature of −80°C.

### Measures

#### PTD outcome

Pregnant women self-reported their last menstrual period (LMP) at the 1^st^ prenatal care visit (usually at 8–10^th^ week of gestation). The LMP was confirmed by early ultrasound assessment (gestational age<20 full weeks). If self-reported LMP was unavailable, ultrasound estimated LMP based on the crown-rump length in early pregnancy was used instead [25]. We calculated gestational age as the interval between LMP and delivery date. We defined PTD as gestational age less than 37 complete weeks.

#### Passive smoking exposures

In this study, we measured maternal passive smoking during pregnancy by combining self-report and serum cotinine test. At the interview, participants retrospectively self-reported their passive smoking status during pregnancy.

We also measured the cotinine level in maternal peripheral blood using enzyme immunoassay technique (Immunalysis Corp., Pomona, California, US; Manufacture-reported detection limit, 1 ng/ml). Briefly, we first added 10 µl of serum, calibrator, or control to 3 separate assay wells, and mixed them with 100 µl of cotinine enzyme. Then we incubated these samples for 30 minutes at room temperature, and washed the micro-plate 6 times with 350 ul buffer. Then we added 100 µl of substrate solution and measured the absorbance spectrum of samples at 450 nm using a micro-plate reader within 60 minutes.

We defined passive smoking as: 1) self-reported exposure to cigarette smoke by others (at home, work or public places) during pregnancy (n = 199), or 2) serum cotinine level ≥3 ng/ml (n = 230). This combined use of self-report and serum cotinine could reduce misclassification of passive smoking [26], [27]. Overall, there was relatively high concordance between self-reported passive smoking when serum cotinine cut-off level was set as 3 ng/ml in our sample (Kappa-value: 0.752). However, 53 women (7.3%) who did not report passive smoking but had serum cotinine level ≥3 ng/ml and 22(3.0%) women who reported passive smoking but had serum cotinine level <3 ng/ml (Table S1).

#### Genotypes

We purified DNA from venous whole blood samples using DNS purification kits (Takara, Biot. Ltd, China).We used polymerase chain reaction (PCR) and restriction fragment length polymorphism (RFLP) methods to genotype CYP1A1 polymorphisms. We digested PCR products by Msp I (to identify CYP1A1 m1 mutation) and BsrD I (to identify CYP1A1 m2 mutation) and then detected the genotypes of our interest, including the homozygous wild type “TT” “AA”, heterozygous variant “TC” “AG”, and homozygous variant “CC” “GG”. The PCR primers (Sangon biotech, shanghai, co., Ltd.) for CYP1A1 polymorphisms included Msp I forward 5′-CAG TGA AGA GGT GTA GCC GCT-3′ and reverse 5′-TAG GAG TCT TGT CTC ATG CCT-3′; BsrD I forward 5′-CTG TCT CCC TCT GGT TAC AGG AAG C-3′ and reverse 5′-TTC CAC CCG TTG CAG CAG GAT AGC C-3′. The PCR primers for GSTs polymorphisms included GSTM1 forward 5′-GAA CTC CCT GAA AAG CTA AG-3′ and reverse 5′-GTT GGG CTC AAA TAT ACG GTG G-3′; and GSTT1 forward 5′-TTC CTT ACT GGT CCT CAC ATC TC-3′ and reverse 5′-TCA CCG GAT CAT GGC CAG CA-3′. As the internal control, a 268-bp fragment of the human –β globin gene was coamplified with a second set of primers (5′-CAA CTT CAT CCA CGT TCA CC-3′) and (5′-GAA GAG CCA AGG ACA GGT AC-3′). Due to small numbers of participants with homozygous variant genotypes, we combined TC (n = 310) and CC (n = 127) as TC/CC, AG (n = 266) and GG (n = 71) as AG/GG. For the same reason, we combined GSTM1 and GSTT1 genotypes as a binary variable GSTs that was coded as “null” if both GSTM1 and GSTT1 were null and as “present” otherwise.

#### Confounders

In addition to the matched delivery date, we considered family income, maternal age, education level, and prepregnancy body mass index (BMI) as potential confounders. We classified self-reported family income as low (<1500 Renminbi [RMB, Chinese currency] monthly), middle (1500–4000 RMB monthly), and high (>4000 RMB monthly). We calculated prepregnancy BMI as self-reported prepregnancy weight in kg/height in meter^2^, and classified women into underweight (BMI<18.5), normal (BMI 8.5–24.0), and overweight or obese (BMI≥24.0) according to WHO guideline for Asians [28].

### Statistical Analyses

We first conducted Chi-square/*t*-test to examine the overall characteristic balance between PTD case and control groups. Chi-square test was adopted to test the associations between maternal passive smoking during pregnancy, single gene polymorphism with the risk of PTD, we then fitted multivariable logistic regression models to estimate the odds ratios (OR) and their 95% confidence intervals (CI), adjusting for potential confounders.

To examine whether the association between maternal passive smoking and PTD could be modified by maternal CYP1A1 and GSTs polymorphisms, we tested their interactions on multiplicative scale. Specifically, we added the interaction terms “passive smoking×gene polymorphism” (products) to the multivariable logistic regression models which already included the main effect terms for passive smoking and CYP1A1 and GSTs genotypes, as well as the potential confounders. A significant departure of the OR value for an interaction term from 1 indicated the existence of interaction on a multiplicative scale. To better demonstrate joint gene-passive smoking interaction, we also classified participants into 8 exclusive groups by passive smoking status (yes vs. no), CYP1A1 (“wild” vs. “variant”), and GSTs (“present” vs. “null”). We set non-passive smoking mothers with low risk genotypes (CYP1A1 “TT” or “AA”+GSTs “present”) as the reference group and then compared it with the other 7 groups. All analyses were completed in SPSS 16.0 software (SPSS Inc, Chicago, Illinois, USA). The statistical significance level was set to <0.05 (two-sided).

## Results

Table 1 shows the characteristics of our study sample. Overall, the PTD case and control groups were comparable in terms of maternal age, marital status, socio-economic status, alcohol use during pregnancy, prepregnancy BMI, and the child gender. However, mothers in the case group had lower education level (36.9% vs. 46.9% with college or higher degree), and were also more likely to be parous (32.3% vs. 24.0%), when compared with mothers in the control group.

Table 2 shows the overall associations (main effect) of maternal passive smoking during pregnancy and single gene polymorphism with risk of PTD. The proportion of passive smoking mothers in the PTD group (49.0%) was much higher than those in the control group (29.6%), and the adjusted OR was 2.20 (95% CI: 1.56–3.12). However, the distribution of CYP1A1 Msp I, CYP1A1 BsrD I, GSTM and GSTT1 genotypes were similar between the two groups.

Table 3 shows the interactions between maternal passive smoking during pregnancy and single gene loci polymorphism on risk of PTD, after adjusting for potential confounders. Overall, there was no significant interaction between maternal smoking during pregnancy with any of the 4 selected gene loci (CYP1A1 Msp I, CYP1A1 BsrD I, GSTM1 and GSTT1) on multiplicative scale.

Table 4 and 5 show the interactions between maternal passive smoking during pregnancy and joint gene polymorphisms on risks of PTD, after adjusted for potential confounders. Compared with non-passive smoking women with CYP1A1 “TT” and GSTs “present”, those passive smoking women with CYP1A1 “TC/CC”+ GSTs “null” had higher risk of PTD (OR = 4.72 [95% CI: 2.28–9.77]), the interaction between passive smoking and CYP1A1 “TC/CC”+ GSTs “null” was statistically significant (OR = 2.66 [95% CI: 1.19–5.97; P-value: 0.017]) on multiplicative scale (Table 4). Compared with non-passive smoking women with CYP1A1 “AA” and GSTs “present”, those passive smoking women with CYP1A1 “AG/GG”+ GSTs “null” had higher risk of PTD (OR = 7.01 [95% CI: 2.91–16.86]), the interaction between passive smoking and CYP1A1 “AG/GG”+ GSTs “null” was statistically significant (OR = 3.00 [95% CI: 1.17–7.74; P-value: 0.023]) on multiplicative scale (Table 5).

## Discussion

In this case-control study among Chinese women, we examined the interactions between maternal passive smoking during pregnancy and genes (CYP1A1, GSTs) polymorphisms on the risks of spontaneous preterm delivery. We confirmed that maternal passive smoking was associated with higher risk of spontaneous preterm delivery. In addition, this increased risk was more striking for those women with the jointed genotype of CYP1A1“AG/GG”+GSTs“null”.

We found that maternal passive smoking during pregnancy was associated with more than two-fold risk of PTD, after adjusting for a series of potential confounders. This estimate is very close to a previous study (OR = 2.3[95% CI: 1.96–5.96]) [29]. Our finding adds to the literature that maternal passive smoking during pregnancy is a risk factor for PTD among women [30], [31], [32], [33]. This suggests that pregnant women should try their best to avoid passive smoking, which is especially important in developing societies, such as China where 53% males (probably including their husbands) are daily smokers [4].

There were limited evidences on interaction between CYP1A1, GSTs polymorphisms and maternal active smoking during pregnancy (no study on passive smoking so far) on the risk of PTD. Our study showed that CYP1A1 m1 (Msp I) and m2 (BsrD I) mutation and GSTs deletion alone, in the absence of maternal passive smoking during pregnancy, did not increase the risk of PTD. However, we found a significant synergy between maternal passive smoking and CYP 1A1 “AG/GG” + GSTs “null” genotype, as well as between maternal passive smoking and CYP1A1 “TC/CC” + GSTs “null” genotype; and noted that we defined GSTs “null” if both GSTT1 and GSTM1 are null to maximize the impact of GSTs null function. This suggests these genetic risk factors may amplify the high risk of PTD associated with passive smoking during pregnancy.

Preterm delivery is a complex phenotype with various pathophysiological pathways, and more than 30 single nucleotide polymorphisms (SNPs) have been found to be associated with PTD or premature rupture of the membranes [34], [35]. CYP1A1 and GSTs genes are two of many pathways that control the conversion of exogenous exposure [6]. So, it is likely that PTD cannot be explained by the variation of single gene locus [6]. This may be why we did not find interaction of passive smoking with single loci of gene mutations (CYP1A1 or GSTs). However, we did find significant interaction between joint genotypes of CYP1A1+GSTs and passive smoking, which suggests CYP1A1 combined with GSTs presents a stronger pathophysiological pathway through which passive smoking increases the risk of PTD. This is biologically plausible, because passive smoking mothers with high-risk genotypes (i.e. CYP1A1 “AG/GG” or “TC/CC”) may have the higher-activity enzymes that metabolize cigarette toxins such as PAHs, when GSTs detoxification function is null, higher levels of PAH-DNA adducts and DNA strand breakage were produced and accumulated in the maternal body. Moreover, the activated adducts can cause the placental inflammatory reaction and initiate the uterus contraction thus lead to PTD [36]. Our finding is consistent with a previous study on maternal active smoking done by Tsai et al. [17]. They found a very strong interaction between maternal active smoking and the joint genotypes (CYP1A1“AG/GG”+GSTT1 “null”) in the risk of PTD accompanied by histologic chorioamnionitis.

### Study Strengths and Limitations

This study has several notable strengths. This is the first study on interaction between maternal passive smoking during pregnancy and maternal metabolism genes (e.g. CYP1A1 and GSTs) on the risk of PTD. Secondly, our combined use of self-report and serum cotinine level can largely reduce misclassification of maternal passive smoking due to recall bias and/or biomarker measurement error. Finally, we excluded women with chronic disease and medical induced PTD, which allowed us to more accurately estimate the impact of passive smoking on spontaneous PTD as well as its interaction with CYP1A1 and GSTs genes. However, some limitations should be mentioned. Firstly, the case-control design of our study could only provide suggestive but not confirmative causality regarding the association between maternal smoking during pregnancy and PTD. Secondly, there was substantial uncertainty in our estimated associations due to the relatively small sample size of PTD cases. Thirdly, although we adjusted for maternal socio-demographics and prepregnancy BMI, we could not control some other important confounders, such as air pollution during pregnancy which could increase the risk of preterm birth [37], [38]. Finally, we could not distinguish the timing (e.g. by trimester) of passive smoking during pregnancy.

### Conclusion

In summary, we found maternal passive smoking during pregnancy was associated with higher risk of spontaneous PTD. The genotypes of CYP1A1 “AG/GG” or “TC/CC” + GSTs “null” seemed to amplify the risk of spontaneous PTD associated with passive smoking during pregnancy. This novel finding has important clinical and public health implications. It not only contributes to better understanding the pathogenic pathways through which maternal passive smoking increases risk of spontaneous PTD, but also helps to identify vulnerable pregnant women who are subject to high risk of spontaneous PTD due to maternal passive smoking.

## Supporting Information

Table S1 **Concordance of maternal passive smoking measured by self-report and by maternal serum cotinine level.**
(DOC)
