# Supplementary material for: Interaction between Maternal Passive Smoking during Pregnancy and CYP1A1 and GSTs Polymorphisms on Spontaneous Preterm Delivery
Source: PLoS One. 2012 Nov 13;7(11):e49155. doi: 10.1371/journal.pone.0049155 (PMC3496734; doi:10.1371/journal.pone.0049155)
Supplement: Table S1 — Concordance of maternal passive smoking measured by self-report and by maternal serum cotinine level. (DOC) [file pone.0049155.s001.doc]

**Supporting information**

**Table S1 Concordance between maternal passive smoking measured by self-report and by different cut-off points of** maternal serum cotinine level

| **Cut-off point of maternal serum cotinine level (ng/ml)** | | **Self-report** | | **Kappa** |
| --- | --- | --- | --- | --- |
|  |  | **Non-exposure**  **N.(%)** | **Passive smoking**  **N.(%)** |  |
| ≥1.0 | Non-exposure | 354(49.0) | 4(0.6) | 0.523 |
|  | Passive smoking | 169(23.4) | 195(27.0) |  |
| ≥2.0 | Non-exposure | 417(57.8) | 13(1.8) | 0.639 |
|  | Passive smoking | 106(14.7) | 186(25.8) |  |
| ≥3.0 | Non-exposure | 470(65.1) | 22(3.0) | 0.752 |
|  | Passive smoking | 53(7.3) | 177(24.5) |  |

% indicates percent within the 2 by 2 table for each cut-off point of serum cotinine level.
